# Live related donor lobar lung transplantation recipients surviving well over a decade: still an option in times of advanced donor management

**DOI:** 10.1186/1749-8090-8-37

**Published:** 2013-03-07

**Authors:** Prashant N Mohite, Aron F Popov, Magdi H Yacoub, Andre R Simon

**Affiliations:** 1 Department of Cardiothoracic Transplantation & Mechanical Support, Royal Brompton and Harefield NHS Trust, Harefield Hospital, Hill End Road, Harefield, UB9 6JH, Middlesex, UK; 2 Harefield Heart Science Centre, National Heart and Lung Institute, Imperial College London, Harefield Hospital, UB9 6JH, London, UK; 3 Postal Address: Harefield Hospital, UB9 6JH, Middlesex, UK; 4 Georg-August-University Goettingen, Robert-Koch-Str. 40, D - 37075, Goettingen, Germany

**Keywords:** Live related donor lobar lung transplantation, Live donor lobar lung transplantation, Lung transplantation

## Abstract

As waiting lists for lung transplantation are ever increasing, the number of organ donors is not able to keep pace with it. Living donor lobar lung transplantation is a source of organs which could be lifesaving in end-stage lung disease patients who cannot wait for cadaveric organs due to deteriorating lung function and clinical condition. Two young women with end stage cystic fibrosis received lobes from their relatives and an altruistic friend. They are surviving for more than 12 and 14 years with good lung functions.

## Background

Living donor lobar lung transplantation (LDLLT) is performed as a life-saving procedure for critically ill patients who are unlikely to survive the long wait for cadaveric lungs. It has been proved life saving for various lung diseases and appears to provide similar or better survival than cadaveric lung transplantation. We are reporting two LDLLT recipients surviving for more than a decade with good lung functions.

## Case presentation

### Case 1

**Figure 1:**
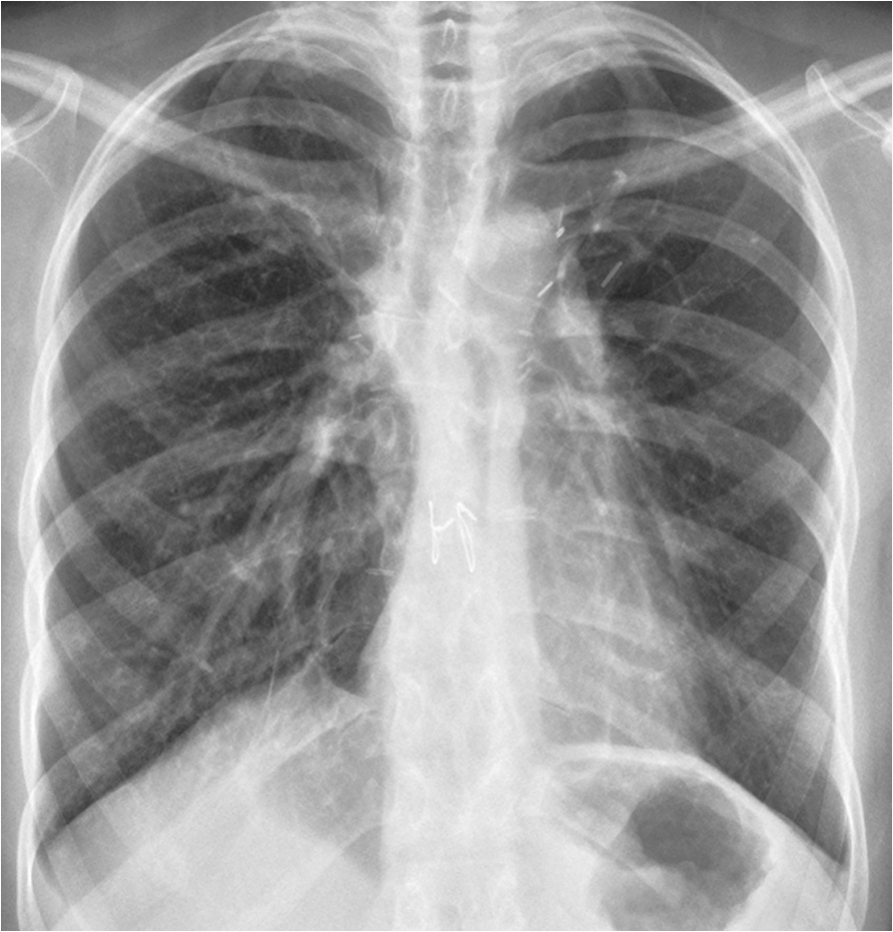
Chest x-ray in case 1.

A 25 years old university graduate student diagnosed of cystic fibrosis with pancreatic insufficiency in childhood was started deteriorating clinically due to uncontrolled infection in chest, not responding to multiple courses of various intravenous antibiotics over 6 months. Her main complaints were cough with copious green expectorations and shortness of breath with which she could barely manage her household work. After detailed clinical examination and investigations, she was accepted on the waiting list for lung transplantation. Following repeated respiratory infections, her lung function deteriorated rapidly (Forced vital capacity, FVC 28% and forced expiratory volume in 1 s, FEV_1_ 25%) and she became bed ridden. As it became apparent that her clinical condition was falling so fast that she will not survive a waiting for suitable cadaveric donor lungs, her family was given a choice of live related lobar lung transplantation, for which it agreed. The donors were her biological father, aged 60 years (Height: 182 cm, weight: 87 kg) and real sister, aged 24 years (Height: 176 cm, weight: 65 kg). The recipient height and weight were 164 cm and 53 Kg. The recipient, the right lobar donor (father) and the left lobar donor (sister) were taken to the different theatres with gap of half to one hour each, in that order. The recipient had clamshell incision and both the lungs were dissected. She was put on cardiopulmonary bypass (CPB) and right pneumonectomy was performed. The right lower lobectomy was performed in father through right postero-lateral thoracotomy at the same time and the lobe was brought to recipient theatre. The bronchial anastomosis (4–0 PDS, continuous) was followed by pulmonary artery (6–0 polypropelene, interrupted) and pulmonary venous (6–0 polypropelene, continuous) anastomoses. The left pneumonectomy was performed in the recipient and the left lower lobe was obtained from the sister and anastomosed in similar fashion. The ischemic time for right side was 2 hours while for left side it was 1 hour and 5 minutes. The recipient was weaned off CPB with modest inotropic support and nitric oxide and blood gases were suggestive of good gas exchange. Immunosuppression protocol in LDLLT was similar to cadaveric donor lung transplantation, consisting of tacrolimus, mycophenolate mofetil and prednisolone and was started in the patient on the day of transplantation. Recipient was weaned off ventilator in 48 hours, but had slow recovery and long intensive care unit stay due to infection with Pseudomonas, persistent pleural effusions, renal failure needing intermittent hemofiltration for a month and early rejection requiring re-intubation and methyl prednisolone pulse. She was discharged home 3 months after surgery and over another 6 moths her lung functions and exercise tolerance kept improving steadily. Over the last 12 years her respiratory function tests have fallen twice but bronchial biopsy never showed rejection. Her latest lung function showed predicted FVC of 65% and predicted FEV_1_ of 66%. Figure 1 shows her latest chest x-ray. She is full time, efficient office worker. Post-operative period was uneventful for both the donors who made fast and excellent recovery and were discharged home on 8^th^ day.

### Case 2

**Figure 2:**
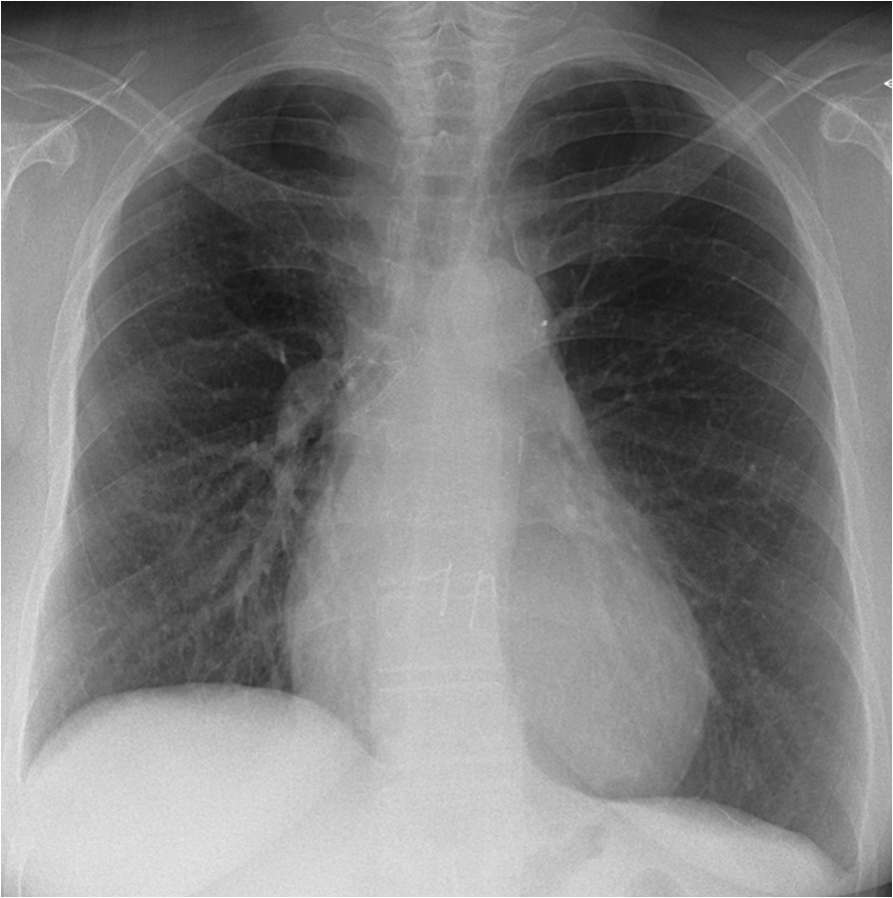
Chest x-ray in case 2.

Another woman of 34 years with cystic fibrosis related end stage lung disease had complaints of cough, haemoptysis, dyspnoea, early fatigue and was requiring nocturnal oxygen therapy. Her clinical condition and lung function deteriorated rapidly following several repeated bouts of respiratory infections. Implied, that it was difficult for her to wait for cadaveric donor organs, her family was given option of LDLLT. As her mother, who already agreed to donate a lobe was only close relative alive; her altruistic friend expressed a wish to donate a lobe. She (Height: 164 cm, Weight: 50 kg) received a right lower lobe from a 24 years old altruistic friend (Height: 164 cm, Weight 72 kg) and a left lower lobe from her biological mother, aged 54 years (Height: 154 cm, Weight: 80 kg) through a clamshell incision in a way mentioned in case 1. The ischemic time for right side was 2 and half hours while for left side it was 1 hour and 20 minutes. The patient was weaned off ventilator on third day; however her post-operative course was complicated by fungal endophthalmitis requiring right side evisceration. She was discharged after 2 months. At the end of one year, she developed collapse of the right lung due to stenosis of right bronchial anastomosis which required endobronchial stenting. Her latest lung function showed predicted FVC of 78.6% and predicted FEV_1_ of 84% and she is leading a healthy life. Figure 2 shows her latest chest x-ray. Post-operative period was uneventful for both the donors, the friend was discharged on 5^th^ while the mother on 7^th^ day.

## Discussion

Lung transplantation is now established as a treatment option for end-stage pulmonary disease [1]. The demand for organs is ever increasing and far exceeds the supply. The number of suitable organ donors could be increased by proper donor management, non-heart beating donor, ex-vivo lung perfusion and LDLLT [2,3]. Usually, LDLLT is a last option chosen to save critically ill patient with end-stage pulmonary disease who cannot wait for organs from cadaveric donor [4]. In the present cases, the patients had cystic fibrosis and had rapid deterioration of their lung function which left us without any choice but to offer them option of LDLLT with relatives as donors.

The ethical dilemma in LDLLT is whether family members should be risked in order to save a relative [5]. Two lungs obtained from live donors can adequately support an adult cystic fibrosis patient [6]. In our first case, donors were 7–10% taller compared to recipient, while in the second case they were equal or shorter than the recipient. However, donor lower lobes filled up recipient hemithorices adequately. LDLLT is severely limited by availability of suitable wishful donor in the family. Most important criteria to be met is suitably matching blood group. If more than one wishful donor in family meets this criteria, then height, weight and age matching comes in picture. Size mismatching can be overcome to a certain extent using various surgical techniques, however they were not required in the present cases [4]. Although LDLLT may be associated with the limitation of size mismatch, it holds promise for providing well-functioning pulmonary lobar grafts to critically ill patients with poor life expectancy [7]. The donor procedure is safe with minimum morbidity, well tolerated physiologically, and the great majority of donors are extremely satisfied with their decision to donate [6,8]. In two recent large LDLLT series, there was no mortality in live lobar donors and 15–20% donors suffered some kind of morbidity which is acceptable and similar to the standard lung resection; moreover donor pulmonary function was found well preserved [9,10]. In present cases, the donors made excellent recovery without any complication and are experiencing healthy lifestyle more than a decade after surgery. LDLLT provides acceptable long-term survival when compared to recipients of cadaveric grafts [8]. This could possibly be because of avoidance of organ transport on ice, thus preserving the grafts structurally and functionally as well as less chance of rejection, probably because of sharing genetic pool with the donors. In our first case, the donors were recipient’s first relatives and she never had biopsy proven rejection in any of the donated lobes. After 12 years of transplantation, she is following up with good lung function tests and having near-normal lifestyle. In our second case, although one of the donors was not related to the recipient biologically, she never had biopsy proven rejection and shown consistently good lung function since 14 years of transplantation.

In case of lung transplantation, the breathing capacity and exercise tolerance increases initially after surgery, then plateau and after 5–7 years and then starts decreasing as transplanted lungs inevitably develop bronchiolitis obliterans. Interestingly, in both our LDLLT recipients lung function has improved over time and recipients feel that the breathing and exercise capacity has increased over the years and it was always better than before. These two cases do not represent our institute’s experience of LDLLT, but embodies good outcome and long term survival in patients undergoing LDLLT.

## Conclusions

LDLLT is a source of organs which could be life saving in end-stage lung disease patients who are likely to die on list waiting for cadaveric organs. As the procedure involves risk to healthy donors, proper assessment of family members as a donor, appropriate recipient-donor size matching and superlative timing of recipient-donor surgeries is a key to success. Although cadaveric donors remain the main source of organs, LDLLT should continue to be used under properly selected circumstances, to maintain the viability of this potentially life-saving procedure.

## Consent

Written informed consent was obtained from the patients for publication of this Case report and accompanying images. A copy of the written consent is available for review by the Editor-in-Chief of this journal.

## Abbreviations

LDLLT: Living donor lobar lung transplantation; FVC: Forced vital capacity; FEV1: Forced expiratory volume in 1 second; CPB: Cardiopulmonary bypass

## Competing interests

The authors declare that they have no competing interests.

## Authors’ contributions

PNM drafted the manuscript, AFP analyzed and interpreted the patient data, YM performed the surgery and was a major contributor in writing the manuscript, ARS conceptualize the manuscript and was involved in the crucial revisions of manuscript. All authors read and approved the final manuscript.

## References

[B1] Trulock EP Christie JD Edwards LB Boucek MM Aurora P Taylor DO Dobbels F Rahmel AO Keck BM Hertz MI Registry of the international society for heart and lung transplantation: twenty-fourth official adult lung and heart-lung transplantation report-2007 J Heart Lung Transplant 2007 26 8 782 795 10.1016/j.healun.2007.06.003 17692782

[B2] Yeung JC Cypel M Waddell TK van Raemdonck D Keshavjee S Update on donor assessment, resuscitation, and acceptance criteria, including novel techniques-non-heart-beating donor lung retrieval and ex vivo donor lung perfusion Thorac Surg Clin 2009 19 2 261 274 10.1016/j.thorsurg.2009.02.006 19662970

[B3] Aigner C Winkler G Jaksch P Seebacher G Lang G Taghavi S Wisser W Klepetko W Extended donor criteria for lung transplantation–a clinical reality Eur J Cardiothorac Surg 2005 27 5 757 761 10.1016/j.ejcts.2005.01.024 15848310

[B4] Date H Update on living-donor lobar lung transplantation Curr Opin Organ Transplant 2011 16 5 453 457 10.1097/MOT.0b013e32834a9997 21836512

[B5] Kramer MR Sprung CL Living related donation in lung transplantation. Ethical considerations Arch Intern Med 1995 155 16 1734 1738 10.1001/archinte.1995.00430160054006 7654106

[B6] Barbers RG Cystic fibrosis: bilateral living lobar versus cadaveric lung transplantation Am J Med Sci 1998 315 3 155 160 10.1097/00000441-199803000-00004 9519928

[B7] Yamane M Date H Okazaki M Toyooka S Aoe M Sano Y Long-term improvement in pulmonary function after living donor lobar lung transplantation J Heart Lung Transplant 2007 26 7 687 692 10.1016/j.healun.2007.04.008 17613398

[B8] Bowdish ME Barr ML Starnes VA Living lobar transplantation Chest Surg Clin N Am 2003 13 3 505 524 10.1016/S1052-3359(03)00058-9 13678310

[B9] Bowdish ME Barr ML Schenkel FA Woo MS Bremner RM Horn MV Baker CJ Barbers RG Wells WJ Starnes VA A decade of living lobar lung transplantation: perioperative complications after 253 donor lobectomies Am J Transplant 2004 4 8 1283 1288 10.1111/j.1600-6143.2004.00514.x 15268729

[B10] Chen F Fujinaga T Shoji T Sonobe M Sato T Sakai H Bando T Date H Outcomes and pulmonary function in living lobar lung transplant donors Transpl Int 2012 25 2 153 157 10.1111/j.1432-2277.2011.01401.x 22187975

